# Inspiring the Study of Gerontology Among Millennial Students: A Rural Perspective

**DOI:** 10.1093/geroni/igaa057.044

**Published:** 2020-12-16

**Authors:** Elaine Jurkowski

**Affiliations:** Southern Illinois University at Carbondale, Saint Charles, Missouri, United States

## Abstract

The study of gerontology across different generational cohorts is not static but rather dynamic. As we consider teaching gerontology to different age cohorts, what similarities and differences do exist? How do the educational needs within higher education differ across the different cohorts of the workforce? This presentation aims to identify and present specific learning needs and styles of the millennial cohort, as compared to other generational cohorts with the effort of building educational strategies for this workforce cohort. The presentation will also address the unique needs that a rural workforce faces and how to address these within the millennial cohort. Strategies such as online learning, flip classroom, experiential strategies and research projects/practicum will be addressed with a specific focus on the engagement of the millennial student. These strategies are all described within the context of teaching gerontology through a specific Certificate in Gerontology program located on a rural campus setting, but meeting the educational needs of people across the country.

